# A case of pancreatic head cancer with Trousseau’s syndrome treated with radical resection and anticoagulant therapy

**DOI:** 10.1186/s40792-023-01585-z

**Published:** 2023-01-12

**Authors:** Takumi Kitahama, Shimpei Otsuka, Teiichi Sugiura, Ryo Ashida, Katsuhisa Ohgi, Mihoko Yamada, Katsuhiko Uesaka

**Affiliations:** Division of Hepato-Biliary-Pancreatic Surgery, Shizuoka Cancer Center, 1007, Shimongakubo, Nagaizumi-Cho, Sunto-Gun, Shizuoka, 411-8777 Japan

**Keywords:** Pancreatic ductal adenocarcinoma, Trousseau’s syndrome

## Abstract

**Background:**

The primary disease of Trousseau’s syndrome is often highly advanced cancer, and treatment of the primary disease after cerebral infarction is often difficult. We herein report a case of pancreatic head cancer with Trousseau’s syndrome treated with radical resection and anticoagulant therapy.

**Case presentation:**

A 78-year-old man was admitted with dizziness and diagnosed with cerebral infarction. Abdominal contrast-enhanced computed tomography for a thorough checkup indicated borderline resectable pancreatic head cancer. Radical resection after neoadjuvant chemotherapy (NAC; gemcitabine plus nab-paclitaxel) was scheduled. During the second course of NAC, multiple cerebral infarctions recurred, and the patient was diagnosed with Trousseau’s syndrome. Continuous intravenous infusion of heparin was started for cerebral infarction. Since it was impossible to continue NAC and there was no worsening of imaging findings, radical resection was planned. Thereafter, he underwent pancreatoduodenectomy with superior mesenteric vein resection. The patient progressed well and was discharged on the 19th day after surgery. He continued subcutaneous injection of heparin at home and is alive without recurrence of cancer or cerebral infarction at more than 21 months after surgery.

**Conclusion:**

Surgical treatment may be an option for pancreatic cancer with Trousseau’s syndrome under favorable conditions.

## Background

Trousseau’s syndrome is a condition characterized by systemic thrombosis associated with hypercoagulability due to an underlying malignancy [1, 2]. It was first reported by Armand Trousseau in 1865 as migratory superficial thrombophlebitis [3]. Currently, the term “Trousseau’s syndrome” is often used in the same sense as “cancer-associated thrombosis” to describe a hypercoagulation disorder in patients with malignancy [4].

Patients with Trousseau’s syndrome often have advanced tumors, and their median survival time is reported to be 84 days [5]. Therefore, most reports of pancreatic cancer with Trousseau’s syndrome have involved unresectable pancreatic cancer [6–8], with no reports of resectable or borderline resectable cases in the English literature.

We herein report a patient with pancreatic head cancer who was diagnosed with Trousseau’s syndrome and treated with radical resection and anticoagulant therapy.

## Case presentation

**Fig. 1:**
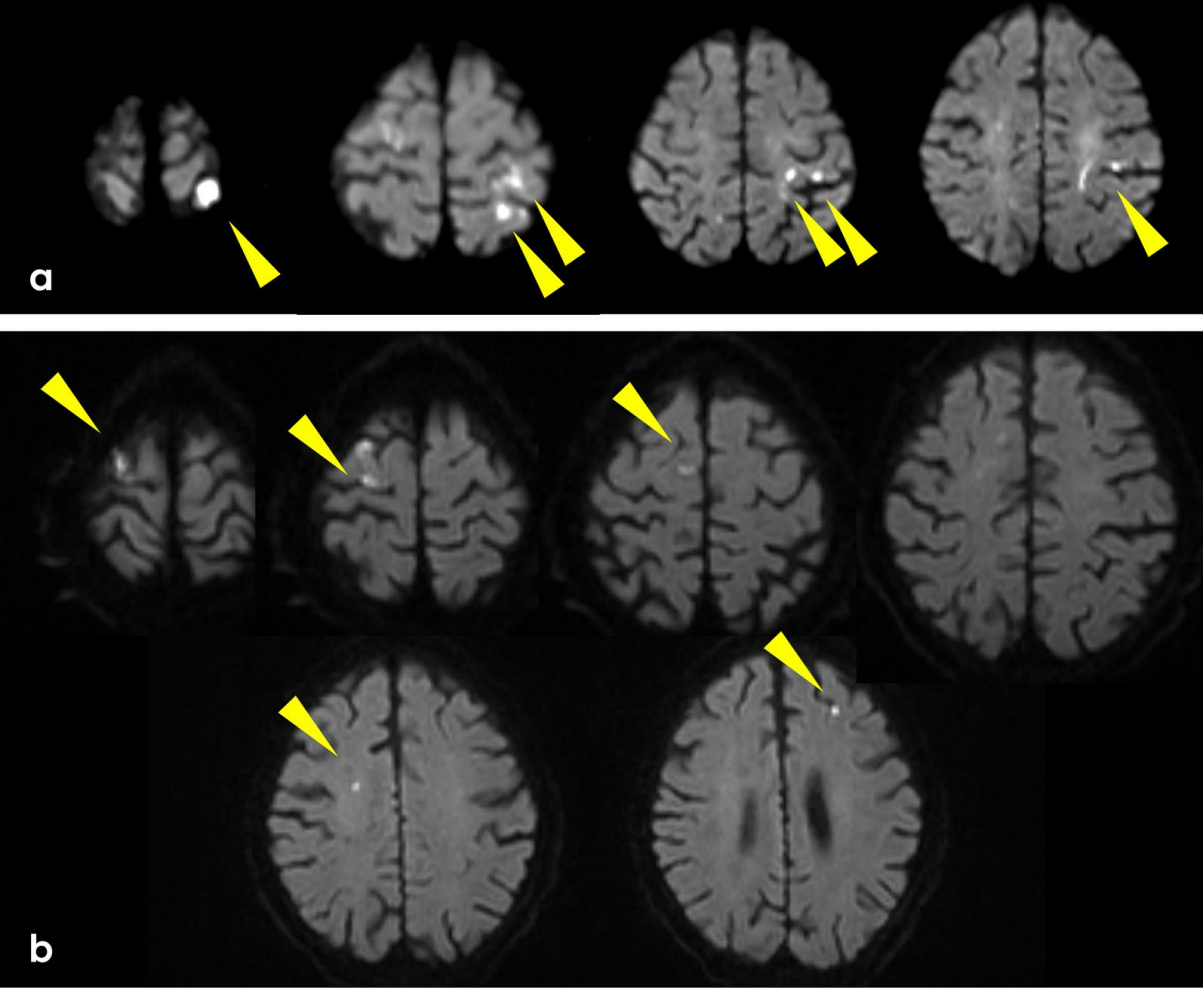
Diffusion-weighted magnetic resonance imaging at **a** the initial cerebral infarction and **b** the second cerebral infarction. **a** Multiple foci of restricted diffusion in the left parietal lobe. **b** Many areas of restricted diffusion in the bilateral lobes

A 78-year-old man developed dizziness, and head magnetic resonance imaging (MRI) revealed multiple infarcted areas in the left parietal lobe (Fig. 1a) at another hospital. His medical history included gastric ulcer at 45 years old and hypertension under treatment. He was diagnosed with cerebral embolism of the left parietal lobe, and treatment with the direct oral anticoagulant (DOAC) dabigatran was started.

Five days after the onset of the cerebral infarction, the dizziness completely recovered. Cardiogenic cerebral embolism was suspected based on the presence of transient atrial fibrillation, but the definite cause of the stroke was unknown. Contrast-enhanced abdominal computed tomography (CT) revealed an ill-defined mass in uncinate process of the pancreas. Fifty days after the onset of cerebral infarction, he presented to our hospital without neurologic findings, and his Eastern Cooperative Oncology Group performance status was 0. He lost weight with 4 kg in the past month. Serum total bilirubin and biliary enzymes, such as aspartate transaminase, alanine transaminase, alkaline phosphatase, and γ-glutamyl transpeptidase were not elevated. Blood coagulation test such as d-dimer, prothrombin time, and activated partial thromboplastin time were all within normal limits.

**Fig. 2:**
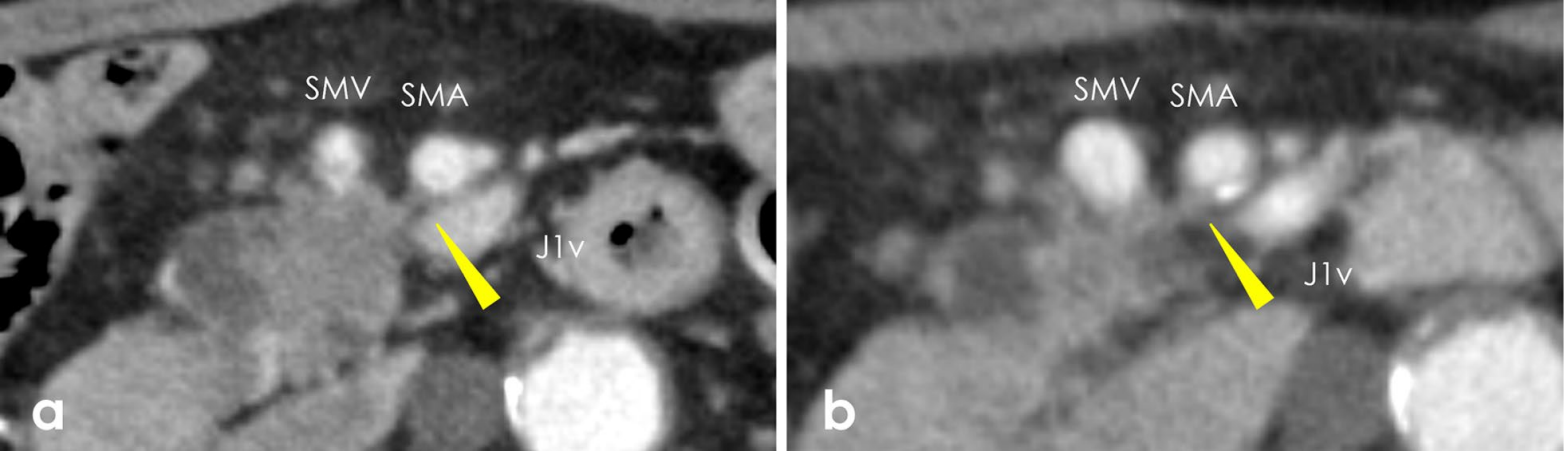
Abdominal contrast-enhanced computed tomography: **a** before neoadjuvant chemotherapy and **b** after neoadjuvant chemotherapy. Arrowheads indicate ≤ 180° of tumor involvement with the superior mesenteric artery. **a** A 24-mm low-density mass was detected at the uncinate process of the pancreas. The tumor involved ≤ 180° of the circumference of the superior mesenteric vein and artery. **b** The tumor shrank to 19 mm in size, and the response to preoperative chemotherapy was stable disease. The classification of tumor resectability was unchanged

Contrast-enhanced abdominal CT at our hospital revealed a 24-mm hypodense mass at the pancreas head invading the superior mesenteric vein (SMV) and superior mesenteric artery (SMA) with abutment ≤ 180º (Fig. 2a). The tumor did not invade common bile duct. There was no evidence of lymph node involvement or visceral metastatic spread. Tumor markers (CEA, CA19-9, DUPAN-2, and Span-1) were within their respective normal ranges. Adenocarcinoma was detected in the pancreatic head mass by endoscopic ultrasound-guided fine-needle aspiration. Based on these findings, the diagnosis was borderline resectable pancreatic adenocarcinoma [9]. Because the clinical course of the cerebral infarction was good with no residual symptoms, we considered the patient likely to tolerate curative treatment including chemotherapy and surgery. Therefore, radical resection after neoadjuvant chemotherapy (NAC; two courses of gemcitabine plus nab-paclitaxel [10]) was planned.

During the second course of NAC, the patient complained of hematochezia, and upper gastrointestinal endoscopy revealed hemorrhagic multiple gastric ulcers. NAC was discontinued, and radical resection was planned at this point. In addition, 5 days later, he developed left hemiplegia and was diagnosed with multiple cerebral infarctions by MRI (Fig. 1b). Serum d-dimer was 3.9 µg/mL, slightly elevated from normal range. Ultrasonography of the carotid arteries, transthoracic echocardiology, and Holter electrocardiogram did not reveal the source of the embolism. Therefore, the patient was diagnosed with Trousseau’s syndrome related to pancreatic adenocarcinoma and we considered that both the first and the second strokes were caused by Trousseau’s syndrome. The anticoagulant was changed from oral dabigatran to intravenous heparin. Continuous intravenous infusion of unfractionated heparin was started in order to maintain an activated partial thromboplastin time (APTT) of 40–50 s.

To re-determine the treatment strategy for pancreatic cancer, contrast-enhanced abdominal CT and [^18^F]-2-fluoro-2-deoxy-d-glucose (FDG)-positron emission tomography (PET)/CT were conducted. CT revealed that the tumor had shrunk from 24 to 21 mm in size, while the tumor abutment to the SMA and SMV was unchanged (Fig. 2b). PET/CT revealed the accumulation of FDG in the tumor and no findings of new distant metastasis. The tumor response to NAC was classified as a stable disease [11]. Tumor markers, including CEA and CA19-9, were still within normal limits. It was difficult to decide on the best treatment for the patient. We discussed our options with the gastroenterologist and neurologist. Finally, after informing the patient and his family of the risks and benefits of radical resection, we obtained their consent and planned the operation.

Pancreatoduodenectomy was performed with combined resection of the SMV on the 176th day after the initial cerebral infarction. Dissection of the right half of the SMA nerve plexus was performed at the point of invasion. The operation time was 403 min, and intraoperative blood loss was 229 g. The resected tumor was 25 × 20 mm in size, and the pathological diagnosis was moderately differentiated pancreatic ductal adenocarcinoma without mucinous component. Although the tumor invaded portal vein, duodenum, posterior peripancreatic tissue, and pancreatic head nerve plexus, the resection margin was free of tumor cells. Microscopic lymphovascular invasion was observed but lymph node metastasis was not detected (pT2pN0M0 pStage IB, UICC 8th). About 10–50% of the tumor cells changed to fibrous tissue, which was defined as grade IIa in the Evans classification [12]. Continuous intravenous heparin was administered until nine hours before the start of surgery and was resumed on postoperative day 2. When heparin resumed, serum d-dimer level was 24.1 µg/mL.

On postoperative day 9, the patient had an elevated d-dimer value (76 μg/mL) and decreased APTT values from therapeutic to normal range (29.5 s) without any symptoms, and contrast-enhanced CT revealed a pulmonary embolism. However, the patient remained asymptomatic with only a heparin dosage adjustment. He was discharged on day 19 after surgery. The administration of heparin was switched from continuous intravenous infusion at the hospital to subcutaneous injection (10,000 U/day) at the patient’s home and has continued to the present. At the time of discharge, serum d-dimer level was improved to 15.4 µg/mL.

**Fig. 3:**
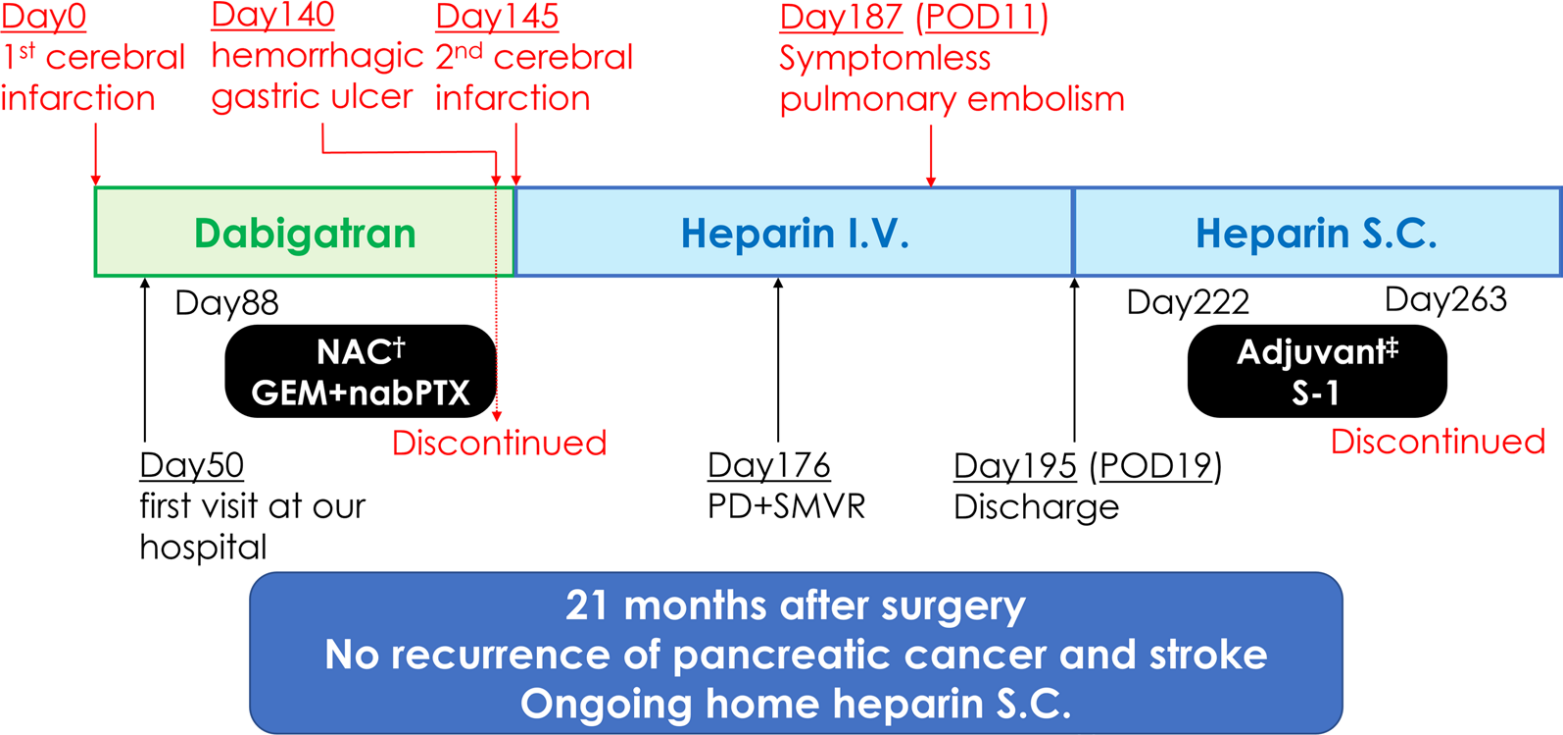
Clinical course. † Ended in the middle of the second course. ‡ Ended after one course due to anorexia. *GEM* gemcitabine, *I.V.* intravenous injection, *PTX* paclitaxel, *PD + SMVR* pancreatoduodenectomy with superior mesenteric vein resection, *POD* postoperative day, *S.C.* subcutaneous injection

As the patient’s general condition was good and subcutaneous heparin injections were safely administered, adjuvant chemotherapy (S-1 80 mg/day) was started 46 days after surgery. However, it was discontinued after one course due to anorexia (Common Terminology Criteria for Adverse Events grade 1 [13]) and poor compliance. The patient is alive at more than 21 months after surgery without recurrence of thrombosis or cancer. The clinical course of the patient is summarized in Fig. 3.

## Discussion

We herein report a case of borderline resectable pancreatic head cancer with Trousseau’s syndrome that successfully underwent radical resection with perioperative anticoagulation therapy. This case demonstrated that radical treatment of pancreatic cancer does not have to be abandoned even if the patient has Trousseau’s syndrome. In this case, a recurrence-free survival of more than 21 months after surgery was achieved for both cancer and cerebral infarction. We therefore believe that the clinical course of this case is worth reporting.

In Trousseau’s syndrome patients, systemic venous and arterial thrombosis is caused by various factors, including activation of coagulopathy by the tumor itself, disseminated intravascular coagulation (DIC), and nonbacterial thrombotic endocarditis (NBTE) [14]. The incidence of embolism varies depending on the histological type of cancer, with adenocarcinoma being 1.65 times more frequent than squamous cell carcinoma [15]. In a report examining 320,000 people [16], pancreatic cancer (3.4%) was second to lung cancer (5.1%) in the 3-month cumulative incidence rates of stroke after the diagnosis of cancer. Especially when it comes to venous thromboembolism (VTE), pancreatic cancer is reported to be the most frequent [17]. Patients who develop Trousseau’s syndrome often have advanced cancer, and the primary disease is often difficult to treat and the prognosis poor; the 1-year relative mortality rate for pancreatic cancer cases diagnosed with Trousseau’s syndrome is over 80% [18]. There are only two cases (non-English literature) of surgical resection for pancreatic cancer with Trousseau’s syndrome (Table 1) [19]. Both involved elderly men. One case was diagnosed with pancreatic cancer before occurring cerebral infarction [19], and the other was diagnosed with pancreatic cancer after cerebral infarction [20], as in the present case. Both had an advanced disease stage and needed extended resection. One patient died of extensive cerebral infarction 2 months after surgery [19], and the other patient survived more than 12 months after surgery, with no cancer recurrence observed [20].

**Table 1:** The cases of resected pancreatic cancer after developing Trousseau's syndrome

Author	Year	Sex	Age	Order of diagnosis	NAC	Tumor location	Surgical Procedure	Combined resection	Anticoagulant after surgery	Outcome (time after surgery)
Nishiwada	2011	M	80	PC → stroke	None	Pb	DP	Celiac axis	Aspirin	Dead by widespread cerebral infarction (3 months)
Hirasawa	2020	M	77	Stroke → PC	Done	Pt	DP	Stomach, transverse colon	Heparin I.V. → Warfarin → suspended	Recurrence (10 months) Alive (16 months)
Present case	2022	M	78	Stroke → PC	Interruption	Ph	PD	SMV	Heparin I.V. → Heparin S.C	Alive without recurrence (15 months)

*DP* distal pancreatectomy, *I.V.* intravenous injection, *NAC* neoadjuvant chemotherapy, *Pb* pancreatic body, *PC* pancreatic cancer, *SMV* superior mesenteric vein, *Ph* pancreatic head, *Pt* pancreatic tail, *S.C.* subcutaneous injection

Trousseau’s syndrome is systemic venous and arterial thrombosis caused by various factors. Although evidence is accumulating concerning the treatment of cancer-associated venous thromboembolism (CAVT) [21, 22], there is no strong evidence regarding cancer-associated arterial thromboembolism (CAAT), as represented by cancer-associated stroke (CAS). Therefore, anticoagulation therapy for patients with Trousseau’s syndrome is often performed according to CAVT. The common treatment for CAVT is low-molecular-weight heparin (LMWH) or vitamin-K antagonist (VKA). However, the 2003 CLOT trial [23] showed the superiority of LMWH over VKA. Since then, LMWH has been considered the first-line treatment for CAVT. LMWH has a short life in blood and requires continuous infusion. In contrast, unfractionated heparin calcium can be administered by subcutaneous injection. Long-term management was reported to be possible with subcutaneous injection of unfractionated heparin, as in the present case [24]. For many years, heparin and VKA were the only options for VTE treatment, but recent clinical trials comparing VKA and DOACs have demonstrated the efficacy of DOACs in cancer patients [25]. DOACs are commonly administered to prevent the development of cardiogenic cerebral embolisms [26]. However, while there are some reports of DOAC usage for Trousseau’s syndrome [27], there are no large-scale reports demonstrating their efficacy for Trousseau’s syndrome. In addition, two large clinical trials comparing aspirin and DOACs for embolic stroke of undetermined source (ESUS) failed to demonstrate the efficacy of DOACs for preventing recurrent stroke over aspirin [28, 29]. Based on these findings, the use of DOACs is not recommended for Trousseau’s syndrome.

In the present case, the patient was able to undergo a pancreaticoduodenectomy with portal vein resection while on perioperative anticoagulation therapy. Heparin was administered continuously except from the period from 9 h before to 24 h after the surgery. Intraoperative blood loss was minimal (229 g), and no bleeding complications were observed postoperatively. Although pulmonary embolism was observed, it was asymptomatic and was able to be followed up with only heparin dosage adjustment. There is no consensus as to how long anticoagulation should be continued after resection of the primary lesion. Due to the existence of various mechanisms underlying the development of Trousseau’s syndrome, continued heparin administration may be acceptable if the situation permits. In the present case, the patient and his family were willing to continue the subcutaneous heparin infusion, understanding the advantages and disadvantages of continued heparin, and the patient had the ability to self-manage his medication with his family willing to provide sufficient support. For these reasons, subcutaneous heparin infusion has been continued since discharge.

This case shows that radical resection need not be abandoned in cases of pancreatic cancer with Trousseau’s syndrome. However, it should be noted that resection is not always recommended for similar cases. The following three points are the rationale for the indication of radical resection in this case: (1) the symptoms of cerebral infarction were completely cured, and the ECOG performance status remained 0; (2) chemotherapy was effective, but it was difficult to continue due to the bleeding gastric ulcer, with no alternative treatment available; and (3) the patient and his family fully understood the possibility of an unwelcome clinical course, such as perioperative bleeding, postoperative early recurrence, and extensive cerebral infarction. These conditions may be helpful when considering surgical treatment of pancreatic cancer with Trousseau’s syndrome. Adjuvant chemotherapy was provided for this case, because the patient’s postoperative recovery was good and subcutaneous heparin was safely administered. The prognosis of pancreatic cancer is poor even after radical resection and the importance of adjuvant chemotherapy is widely known [30]. If the patient’s condition is good, adjuvant chemotherapy should be considered even for the patients with Trousseau’s syndrome.

## Conclusion

We herein report a case of successful radical treatment of pancreatic cancer with Trousseau’s syndrome. If the post-stroke course is good, curative surgery may be an option.

## Data Availability

The data sets supporting the conclusions of this article are included within the article and its Additional files.

## Abbreviations

APTT: Activated partial thromboplastin time
CEA: Carcinoembryonic antigen
CT: Computed tomography
DOAC: Direct oral anticoagulant
FDG: [^18^F]-2-fluoro-2-deoxy-d-glucose
MRI: Magnetic resonance imaging
NAC: Neoadjuvant chemotherapy
PET/CT: Positron emission tomography/computed tomography
PT-INR: Prothrombin time-international normalized ratio
SMA: Superior mesenteric artery
SMV: Superior mesenteric vein
VKA: Vitamin K antagonist
VTE: Venous thromboembolism
